# Serum Glial Cell Line-Derived Neurotrophic Factor (sGDNF) Is a Novel Biomarker in Predicting Cirrhosis in Patients with Chronic Hepatitis B

**DOI:** 10.1155/2022/1048104

**Published:** 2022-07-09

**Authors:** Guangyue Yang, Liping Zhuang, Tiantian Sun, Yee Hui Yeo, Le Tao, Wei Zhang, Wenting Ma, Liu Wu, Zongguo Yang, Yanqin Yang, Dongying Xue, Jie Zhang, Rilu Feng, Ebert Matthias P., Steven Dooley, Ekihiro Seki, Ping Liu, Cheng Liu

**Affiliations:** ^1^Laboratory of Liver Disease, Department of Infectious Disease, Putuo Hospital, Shanghai University of Traditional Chinese Medicine, Shanghai 200062, China; ^2^Experimental Center, Putuo Hospital, Shanghai University of Traditional Chinese Medicine, Shanghai 200062, China; ^3^Department of Integrative Oncology, Fudan University Shanghai Cancer Center, Shanghai, China; ^4^Department of Oncology, Shanghai Medical College, Fudan University, Shanghai 200032, China; ^5^Division of General Internal Medicine, Department of Medicine, Cedars-Sinai Medical Center, Los Angeles, CA 90048, USA; ^6^Department of Integrative Medicine, Shanghai Public Health Clinical Center, Fudan University, Shanghai, China; ^7^Department of Pathology, Putuo Hospital, Shanghai University of Traditional Chinese Medicine, Shanghai 200062, China; ^8^Department of Medicine II, Medical Faculty Mannheim, Heidelberg University, Mannheim, Germany; ^9^Division of Digestive and Liver Diseases, Department of Medicine, Cedars-Sinai Medical Center, Los Angeles, CA 90048, USA; ^10^Institute of Liver Diseases, Shuguang Hospital Affiliated to Shanghai University of Traditional Chinese Medicine, 528 Zhangheng Road, Pudong New District, Shanghai 201203, China

## Abstract

**Objectives:**

We assessed the potential of glial cell line-derived neurotrophic factor (GDNF) as a useful biomarker to predict cirrhosis in chronic hepatitis B (CHB) patients.

**Methods:**

A total of 735 patients from two medical centers (385 CHB patients and 350 healthy controls) were included to determine the association of serum and tissue GDNF levels with biopsy-proven cirrhosis. The diagnostic accuracy of serum GDNF (sGDNF) was estimated and compared with other indices of cirrhosis.

**Results:**

We showed significantly higher levels of sGDNF in CHB patients with fibrosis (28.4 pg/ml vs. 11.6 pg/ml in patients without) and patients with cirrhosis (33.8 pg/ml vs. 23.5 pg/ml in patients without). The areas under receiver operating curve (AUROCs) of sGDNF were 0.83 (95% confidence interval (CI): 0.80–0.87) for predicting liver fibrosis and 0.84 (95% CI: 0.79–0.89) for cirrhosis. Findings from the serum protein level and hepatic mRNA expression were consistent. Using the best cutoff to predict cirrhosis, we categorized the patients into sGDNF-high and sGDNF-low groups. The sGDNF-high group had significantly larger Masson's trichrome and reticulin staining-positive area, higher Scheuer score, and METAVIR fibrosis stage (all *p* < 0.001) but not steatosis. On multivariable regression, sGDNF was independently associated with cirrhosis with an odds ratio of 6.98 (95% CI: 1.10–17.94). Finally, we demonstrated that sGDNF outperformed AST to platelet ratio index, FIB-4, fibroscore, forn index, and fibrometer in differentiating F4 vs. F3.

**Conclusion:**

Using serum, tissue mRNA, and biopsy data, our study revealed a significant potential of sGDNF as a novel noninvasive biomarker for cirrhosis in CHB patients.

## 1. Introduction

Cirrhosis is the leading risk factor for hepatocellular carcinoma and is associated with premature death [1]. Given the high risk of complications from hepatic decompensation, cirrhosis leads to a substantial health burden [2]. Early detection and treatment of cirrhosis may reduce the risk of disease progression and the development of complications. In hepatitis B virus-infected patients, indefinite antiviral treatment is recommended if patients develop cirrhosis [3]. Therefore, early detection of cirrhosis in patients with chronic hepatitis B infection (CHB) is important in informing medical decisions.

Although percutaneous liver biopsy and histological assessment remained the gold standard for diagnosing liver fibrosis [4], the invasiveness limits its wide application [5]. Additionally, the accuracy of hepatic fibrosis assessment is limited by both sampling error and interobserver variability between pathologists. Noninvasive techniques (e.g., serum biomarkers and imaging) are widely performed in countries where these techniques are available and approved [6–8]. Liver stiffness with transient elastography (TE) and magnetic resonance elastography (MRE) are well-validated methods for the assessment of liver fibrosis and cirrhosis [6, 9, 10]. However, these methods are costly and limited to certain liver centers. Moreover, MRE is challenging to perform in some cases, such as for patients with severe iron overload, claustrophobia, or other MR contraindications. Direct serum biomarkers and indirect serum composite scores, such as aspartate aminotransferase to platelet ratio index (APRI), FIB-4 index, and fibrotest are widely used for noninvasive hepatic fibrosis assessment; they are more affordable and can be applied in most clinical settings [11–13]. However, their diagnostic accuracies are limited. Therefore, unmet medical needs for novel biomarkers with better diagnostic performance are significant.

GDNF is a glycosylated, disulfide-bonded homodimer that is a distantly related member of the TGF-*β* superfamily [14]. Clinical studies have found that the GDNF level is increased in the parietal cortex and plasma of recurrent major depressive disorder patients [15]. Additionally, GDNF is increased by several folds following exposure to cytotoxic agents, including radiation [16]. Additionally, GDNF levels are increased in some cancer cell types [17].

Recently, we reported that GDNF promotes hepatic stellate cell activation and liver fibrosis via ALK5/Smad signaling in the preclinical mouse models of liver fibrosis [18]. We also found that hepatic GDNF levels were upregulated in human liver fibrosis [18]. However, the clinical use of GDNF in liver disease remains unclear. In the present study, we assessed GDNF along with biochemical and histological parameters of liver disease in CHB patients. We determined the diagnostic accuracy of serum GDNF in liver fibrosis and cirrhosis and compared that with other known markers.

## 2. Materials and Methods

### 2.1. Ethics Statement

Human samples and study protocol were approved by the Clinical Ethics Committee of Putuo Hospital, Shanghai University of Traditional Chinese Medicine and Shanghai Public Health Clinical Center, Fudan University. The study conforms with the provisions of the Declaration of Helsinki.

### 2.2. Patients

The CHB diagnosis was confirmed by the presence of hepatitis B surface antigen for more than 6 months. A total of 385 CHB patients with serum and biopsy samples and among them, 293 with frozen tissue-derived GDNF mRNA results were included. Serum samples were also obtained from 350 healthy controls who underwent physical examination from December 2017 to July 2019 (Supplemental Figure 1). Serum samples and liver biopsy were collected on the same day from 344 CHB patients at the Putuo Hospital from June 2011 to July 2019. Among them, liver biopsy frozen tissue was procured from 252 patients to determine the liver GDNF mRNA expression. We also collected liver biopsy frozen tissue samples from 41 patients who visited the Shanghai Public Health Clinical Center from November 2013 to March 2016. All patients underwent clinical, biochemical, virological examination, and liver biopsy on the same day. Patients with renal and/or hepatic failure, acute coronary syndromes, valvular heart diseases, autoimmune thyroid diseases, or systematic inflammatory diseases were excluded from our study. Additionally, patients with prior antiviral therapy were excluded.

### 2.3. Histological Liver Fibrosis Staging

Liver biopsy specimens were obtained using 16 G × 20 cm disposable needles (Cat no. MACΠ, Mantova, Italy). The biopsy specimens were then fixed in 4% formalin and embedded in paraffin. Adequate specimens were required to be at least 15 mm in length, and the sections (3 mm thick) were stained with hematoxylin and eosin (HE), reticulin, and Masson's trichrome [19, 20]. The stage of liver fibrosis was scored based on the examination of HE, Masson's trichrome, and reticulin staining by three independent pathologists who were blind to the clinical characteristics of the study subjects at the Putuo Hospital or Shanghai Public Health Clinical Center Department of Pathology. Fibrosis stages were defined based on Scheuer criteria and METAVIR scoring system [20]. According to the Scheuer scoring system, the severity of the liver injury was categorized into G0, G1, G2, G3, and G4, with G1 defined as portal inflammation; G2 as mild piecemeal necrosis; G3 as moderate piecemeal necrosis; G4 as severe piecemeal necrosis and bridging necrosis. According to the METAVIR scoring system, the severity of liver fibrosis was categorized into F0, F1, F2, F3, and F4. F0 was defined as no fibrosis; F1 as portal fibrosis without septa; F2 as septal fibrosis (portal-portal); F3 as septal fibrosis (portal-central); F4 as cirrhosis.

The images of Masson's trichrome and reticulin staining were captured using a BX43 Olympus microscope (Olympus Tokoyo, Japan) and processed using DP73 version software. To quantify Masson's trichrome and reticulin staining, images of five or six randomly chosen fields of each section were taken. The collagen values are expressed as the percentage of the area of the section occupied by Masson's trichrome and reticulin staining using Image-Pro Plus (IPP) software (Media Cybernetics, MA, USA).

### 2.4. Biochemical Analyses

The serum was collected on the same day as the biopsy. The following parameters were assessed: alanine transaminase (ALT), aspartate transaminase (AST), alkaline phosphatase (ALP), gamma glutamyltransferase (GGT), total bilirubin, prothrombin time, international normalized ratio (INR), fasting glucose, albumin, hemoglobin, platelets, leukocytes, triglycerides, haptoglobin, and cholesterol and its components. All samples, including those from the Shanghai Public Health Clinical Center, were determined using standardized assays and methods from the Department of Clinical Laboratory, Putuo Hospital.

Hyaluronic acid (HA), type IV collagen (CIV), laminin (LN), and type III procollagen (PCIII) were assessed using radiometric assays at the Department of Nuclear Medicine, Putuo Hospital. *α*2-macroglobulin was determined by Dian Diagnostics Co. Ltd. (Shanghai, China).

### 2.5. Definition of Indices for Liver Cirrhosis

  APRI: (AST (U/L)/upper normal limit) × 100/platelets (10^9^/L) [21].
  FIB-4 index: age (years) × AST (U/L)/(PLT (10^9^/L)) × (ALT (U/L)^1/2^) [22].
  Fibrometer: −0.007 PLT (G/L) − 0.049 PI (%) + 0.012AST (U/L) + 0.005 *α*2M (mg/dL) + 0.021HA (*μ*g/L) − 0.270 urea (mmol/L) + 0.027 age (yr) + 3.718 [23].
  Forn index: 7.811 − 3.131 × ln (PLT (10^9^/L)) + 0.781 × ln (GGT (U/L)) + 3.467 × ln (age) − 0.014 × (cholesterol (mg/dl) [23].
  Hepascore: *y*/(*y*+1)
 
*y* = exp [4.185818 − (0.0249 × age) + (0.7464 × sex) + (1.0039 × *α*2M g/L) + (0.0302 × HA *μ*g/L) + (0.0691 × bilirubin *μ*mol/L) − (0.0012 × GGT U/L)], male = 1, female = 0 [24].
  Fibrotest: 4.467 × log (*α*2M (mg/dL)) − 1.357 × log Hap (g/L) + 1.017 log GGT (U/L) + 0.0281 × age + 1.737 × log TBil (*μ*mol/L) − 1.184 × apoA-I (g/L) + 0.301 × (sex) − 5.540, male = 1, female = 0 [12, 13].

### 2.6. Statistical Methods

The PASW Statistics software version 23.0 from SPSS Inc. (Chicago, IL, USA) was used for all analyses. The data were expressed as mean ± standard deviation or median (interquartile range) as appropriate. One-way analysis of variance (ANOVA) was used for the comparison of multiple groups, and student's *t*-test was applied to examine the mean differences in normally distributed continuous variables between groups.

The correlation of clinical, biological, and histological factors with cirrhosis was analyzed using stepwise forward multivariable logistic regression. Variables that showed a *p* < 0.05 on univariable logistic regression were selected for multivariable regression.

To determine the diagnostic accuracy of GDNF and other indices, the area under receiver operating characteristic, sensitivity, specificity, positive predictive value (PPV), and negative predictive value (NPV) were calculated. The optimal cutoff of GDNF and all indices were determined using the Youden index. The DeLong test was used to compare the AUROCs of GDNF to several commonly used indices in predicting cirrhosis [10, 25] using Medcalc software version 15.8 (Ostend, Belgium).

For other materials, please see Supplemental materials.

## 3. Results

### 3.1. Patient Characteristics

As shown in Table 1, patients with fibrosis/cirrhosis (METAVIR stage F1–F4, *N* = 318) had higher serum GDNF (sGDNF) levels (28.4 pg/ml (IQR: 26.2, 31.6)) than patients without fibrosis (11.6 pg/ml (IQR: 7.2, 21.1)) (healthy controls and METAVIR stage F0, *N* = 376) (*p* < 0.001). Meanwhile, cirrhotic patients (METAVIR stage F4, *N* = 33) had high sGDNF levels (33.8 pg/ml [IQR: 29.3, 39.4]) as compared with noncirrhotic patients (healthy controls and METAVIR stage F0–F3, *N* = 661) (23.5 pg/ml (IQR: 10.9, 29.5)) (*p* < 0.001) (Table 2). Other patient characteristics are also summarized in Tables 1 and 2.

### 3.2. Evidence for sGDNF-Based Fibrosis and Cirrhosis Prediction

**Figure 1:**
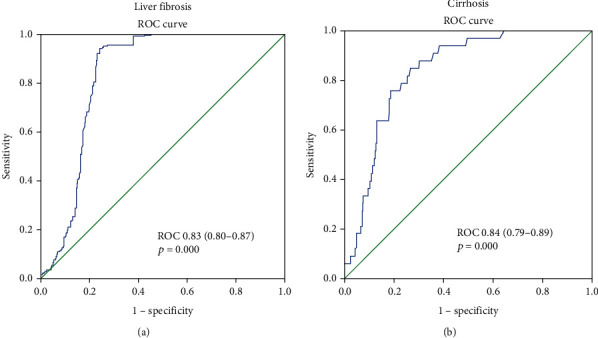
Receiver operating characteristics (ROC) analysis showing the predictive value of sGDNF for liver fibrosis in patients with CHB. Receiver operating characteristic curve for sGDNF predicting liver fibrosis (a) and cirrhosis (b). The estimates indicate the area under the ROC curve.

To investigate the diagnostic accuracy of sGDNF levels for diagnosing liver fibrosis and cirrhosis, we calculated the areas under receiver operating curve (AUROC) values of sGDNF (Figure 1). The AUROCs of sGDNF for diagnosing fibrosis and cirrhosis were 0.83 (0.80–0.87) and 0.84 (0.79–0.89), respectively. Using Youden's index, we determined that the best cutoff value of sGDNF in differentiating cirrhotics and noncirrhotics was 28.74 pg/ml.

### 3.3. Clinical Characteristics and sGDNF

We further categorized the 344 HBV patients into sGDNF-low (<28.74, *N* = 192) and sGDNF-high (≥28.74, *N* = 152) groups (Supplemental Table 1). The sGDNF-high group demonstrated lower serum Alb level (*p*=0.008) as well as higher PCIII (*p*=0.012) and LN (*p*=0.047) values than the sGDNF-low group. There was no significant difference in demographic characteristics, viral load, liver enzymes, and coagulation factors.

### 3.4. Serum and mRNA Levels of GDNF in Different Histopathological Categories

**Figure 2:**
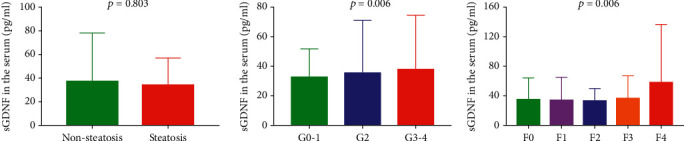
sGDNF levels according to the histological grade and fibrosis stage in 344 patients. Liver histopathology of patients with G0, G1, G2, G3, and G4 according to the Scheuer scoring system. G1, portal inflammation; G2, mild piecemeal necrosis; G3, moderate piecemeal necrosis; G4, severe piecemeal necrosis and bridging necrosis. METAVIR scoring system. F0, no fibrosis; F1, portal fibrosis without septa; F2, septal fibrosis (portal-portal); F3, septal fibrosis (portal-central); F4, cirrhosis.

By examining biopsy samples, we showed that sGDNF-high and sGDNF-low groups did not have significant differences in steatosis (*p*=0.556) (Table 3). Consistently, there were no mean differences in sGDNF levels between subgroups of steatosis (Figure 2). A slight difference in the sGDNF level was found when comparing among G0-1, G2, and G3-4 inflammation stages. Regarding biopsy-proven fibrosis, the sGDNF-high group had higher fibrosis stages (*p* < 0.001), larger reticulin (*p*=0.023), and Masson's trichrome-positive area (*p*=0.004) than the sGNDF-low group (Table 3). Consistently, there was a significantly increasing trend in mean sGDNF levels across the fibrosis stage (Figure 2).

As shown in Supplemental Figure 2, the hepatic mRNA expression of GDNF was in accordance with serum and histopathological examination. Patients with higher G stage and fibrosis stage had significantly higher hepatic mGDNF levels (*p* < 0.001), and there was no significant difference when patients were categorized by steatosis and necrosis stages.

### 3.5. sGDNF as an Independent Factor of Cirrhosis

We conducted a multivariable logistic regression analysis to determine the factors that were associated with cirrhosis (Table 4). After adjusting for a variety of clinical characteristics, cirrhosis was significantly associated with albumin (adjusted odds ratio (aOR) 0.85; 95% CI, 0.74–0.97, *p*=0.019), G stage (aOR 5.55; 95% CI, 1.56–19.67, *p*=0.008), and sGDNF (aOR 6.98; 95% CI, 1.10–17.94, *p*=0.036).

### 3.6. sGDNF Was Superior to APRI, FIB-4, Fibrometer, Forn Index, and Hepascore in Predicting Cirrhosis in CHB Patients

We compared the diagnostic performance of sGDNF with those of APRI, FIB-4, fibrometer, hepascore, forn index, and fibrotest in CHB patients (Table 5). sGDNF (AUROC 0.74 [95% CI, 0.65–0.83]) had a significantly higher AUROC than APRI (0.55 (95% CI, 0.44–0.65); *p*=0.0124), FIB-4 (0.55 (95% CI, 0.46–0.65); *p*=0.0181), fibrometer (0.53; (95% CI, 0.43–0.64); *p*=0.0133), hepascore (0.55; (95% CI, 0.45–0.65); *p*=0.0211), and forn index (0.57 (95% CI, 0.47–0.67); *p*=0.0368), but not fibrotest (0.61 (95% CI, 0.47–0.67); *p*=0.0802) to diagnose F4 vs. F3. No significant difference was noted when comparing the diagnostic accuracy between sGDNF and these indices to diagnose cirrhosis vs. no cirrhosis (F4 vs. F0–3).

## 4. Discussion

In this study, we showed that patients with biopsy-proven cirrhosis and fibrosis demonstrated higher serum protein and tissue mRNA levels of GDNF. A significantly dose-dependent association of the sGDNF level and METAVIR fibrosis stage was observed. sGDNF was associated with significantly higher odds of cirrhosis after adjusting for clinical characteristics. Furthermore, when comparing between the fibrosis stage F3 and F4, sGDNF outperformed most indices for liver cirrhosis including APRI, FIB-4, fibrometer, forn index, and hepascore.

We recently reported that GDNF is the functional promoter of hepatic stellate cell activation and liver fibrosis mediated through ALK5/Smad signaling. We further suggest that GDNF inhibition could be a therapeutic strategy for patients with liver fibrosis [18]. In this study, we observed consistent clinical pictures in CHB patients. We found that sGDNF-high patients have a higher Scheuer score (represented as G stages), larger Masson's trichrome and reticulin staining-positive areas, and higher METAVIR stages. Masson's trichrome staining represents the accumulation of collagen fiber and thus serves as a gold-standard method to diagnose liver fibrosis histologically. Reticulin staining is also a useful tool to stain type III collagen fiber for diagnosing fibrosis severity. Therefore, the results indicated that sGDNF is correlated with inflammation and extracellular matrix production and deposition, which were in line with our previous preclinical studies. Furthermore, the results from sGDNF levels and tissue GDNF mRNA levels were consistent, which enhanced the validation of the findings.

Besides, we also showed that both by serum protein level and tissue mRNA expression in the liver, and GDNF was specifically correlated with liver fibrosis but not with the pattern of necrosis and the existence of steatosis. This exemplified the mechanism of GDNF-mediated liver fibrosis and implied that sGDNF might serve as a powerful noninvasive biomarker for diagnosing fibrosis and cirrhosis. Comparably, on the multivariable logistic regression analysis with an extensive adjustment for clinical confounders, including liver function panels, fibrogenic factors, and blood counts, and sGDNF levels were significantly associated with cirrhosis. Other markers, such as platelet counts, AST, ALT, and total bilirubin levels only showed significance on a univariable but not a multivariable regression.

Multiple studies have used serum markers to predict liver fibrosis. The fibrosis parameters commonly used are related to hepatocyte damage (ALT, AST), macrophages [26], microbiota [27], and hepatic stellate cell activation [28, 29], and these markers could predict advanced fibrosis (F3-4 vs. F0–2) or cirrhosis (F4 vs. F0–3). However, the markers that can predict F4 vs. F3 are uncommon. Given that patients with F3 had a significantly lower risk of hepatic decompensation, hepatocellular carcinoma, overall mortality, and higher transplant-free survival rate, and the clinical relevance to diagnose between F4 vs. F3 is crucially important [30, 31]. Our results show that the sGDNF level can be a first-class predictor for distinguishing F4 cirrhosis from F3 fibrosis. Moreover, comparing sGDNF levels with ARPI, FIB-4, fibrotest, fibrometer, hepascore, and forn index, we found that sGDNF is significantly superior in predicting fibrosis stage F4 vs. F3.

The first strength of this study is that we included patients with biopsy-proven fibrosis/cirrhosis to investigate the correlation between sGDNF levels and the severity of liver fibrosis, especially distinguishing between F4 cirrhosis and F3 fibrosis. Secondly, the results were consistent between serum protein levels and liver tissue mRNA expression of GDNF. Thirdly, we were able to include a large sample size of patients with comprehensive clinical and histological data, which allowed us to minimize residual confounders on multivariable analysis. Fourthly, the pathologists who examined the liver samples were blind to the patients' information.

Moreover, there are limitations to our study. Firstly, we only included CHB patients and thus the results may not be able to be generalized to patients with other chronic liver diseases. However, in our previous preclinical study, we showed that GDNF level was associated with liver fibrosis developed in several different etiologies [18]. Additionally, studies have shown that BRAF can completely affect GDNF-mediated cell survival, and BRAF signaling play a crucial role in the regulation of HCC cell proliferation and survival [32]. GFR*α*1 (GDNF family receptor *α*1), a GPI anchored receptor for GDNF, belongs to the neurotrophic factors (NF) and transforming growth factor-*β* (TGF-*β*) superfamily [33], and has been found predominantly expressed in the liver [34]. GFR*α*1 signaling in promoting invasion, metastasis, and tumor progression has been demonstrated in many different tumor types including glioma and pancreatic cancer [33, 35, 36]. Maybe GDNF can predict the development of HCC. Studies that enroll cirrhotic patients of other primary liver diseases and hepatocellular carcinoma are needed to expand the diagnostic application of sGDNF. Secondly, to enhance the comparability, we only determined the differences between sGDNF levels and other serum marker-based indices. However, we showed that the AUROCs of sGDNF to diagnose cirrhosis (F4) and fibrosis (F1–3) were 0.84 and 0.83, respectively. A previous meta-analysis demonstrated that the pooled AUROCs of acoustic radiation force impulse elastography for examining cirrhosis (F4) and evident fibrosis (≥F2) were 0.93 and 0.85, respectively [37]. Future studies that compare sGDNF with imaging modalities are required because imaging modalities, including MRE and FibroScan, are also useful and accurate in diagnosing liver fibrosis/cirrhosis [38], but they are not as available as serum marker tests in resource-limiting areas and the cost of the test may limit their widespread use.

## 5. Conclusions

In conclusion, we demonstrated that sGDNF is an accurate noninvasive biomarker for diagnosing cirrhosis in CHB patients. The accuracy in differentiating F4 vs. F3 was superior to currently available indices, such as APRI, FIB-4 index, fibrometer, forn index, and hepascore. Validation of its performance in cirrhotic patients of other primary liver diseases is also required.

## Figures and Tables

**Table 1:** Clinical demographics and clinical characteristics of patients in nonfibrosis and fibrosis groups.

	Total (*N* = 694)	Nonfibrosis (*N* = 376)^*∗*^	Fibrosis (*N* = 318)^*∗∗*^	*p* value
Age, yr	43 (34, 55)	45 (39, 62)	40 (32, 48)	0.000
Male, *N* (%)	360 (52)	221 (61)	113 (34)	0.000
ALT, U/L	21 (11, 53)	42 (28, 168)	71 (30, 195)	0.000
AST, U/L	26 (19, 46)	28 (23, 132)	46 (28, 103)	0.000
BUN, mmol/L	4.4 ± 1.3	3.9 ± 1.0	4.7 ± 1.3	0.001
Cr, *μ*mol/L	67 (57, 79)	63 (56, 77)	70 (58, 80)	0.001
RBC, 10^12^/L	4.7 ± 0.5	4.7 ± 0.5	4.8 ± 0.5	0.108
PLT, 10^9^/L	199 ± 61	227 ± 55	183 ± 59	0.000
WBC, 10^9^/L	5.9 ± 3.1	6.4 ± 4.6	5.5 ± 1.5	0.105
Glucose, mmol/L	4.8 (4.4, 5.2)	5.1 (4.7, 5.1)	4.9 (4.5, 5.5)	0.017
sGDNF, pg/ml	25.2 (11.4, 29.5)	11.6 (7.2, 22.1)	28.4 (26.2, 31.6)	0.000

Nonfibrosis is defined by health controls and F0; fibrosis is defined by F1–F4.

**Table 2:** Clinical demographics and clinical characteristics of patients in noncirrhosis and cirrhosis groups.

	Total (*N* = 694)	Noncirrhosis (*N* = 661)^#^	Cirrhosis (*N* = 33)^##^	*p* value
Age, yr	42 (34, 52)	42 (33, 52)	40 (35, 49)	0.605
Male, *N* (%)	360 (52)	335 (51)	25 (76)	0.000
ALT, U/L	21 (11, 53)	16 (10, 51)	107 (53, 448)	0.000
AST, U/L	26 (19, 46)	23 (19, 40)	99 (47, 376)	0.000
BUN, mmol/L	4.7 ± 1.5	4.7 ± 1.5	4.7 ± 1.3	0.858
Cr, *μ*mol/L	67 (57, 79)	79 (67, 88)	67 (60, 80)	0.374
RBC, 10^12^/L	4.7 ± 0.5	4.7 ± 0.5	4.5 ± 0.5	0.021
PLT, 10^9^/L	199 ± 61	203 ± 60	141 ± 42	0.000
WBC, 10^9^/L	5.9 ± 3.0	5.9 ± 3.1	4.9 ± 1.5	0.001
Glucose, mmol/L	4.8 (4.4, 5.2)	4.7 (4.4, 5.2)	4.9 (4.4, 5.5)	0.462
sGDNF, pg/ml	25.2 (11.4, 29.5)	23.5 (10.9, 29.5)	33.8 (29.3, 39.4)	0.000

Noncirrhosis is defined by health controls and F0–F3; cirrhosis is defined by F4. The data are expressed as mean ± standard deviation or median (25%–75%). ALT, alanine aminotransferase; AST, aspartate aminotransferase; BUN, blood urea nitrogen; Cr, creatinine; PLT, platelet count; RBC, red blood cell; sGDNF, serum GDNF; WBC, white blood cell.

**Table 3:** Characteristics of study participants relative to their sGDNF levels.

	Total (*N* = 344)	sGDNF-low (*N* = 192)	sGDNF-high (*N* = 152)	*p* value
Steatosis, *N* (%)				
Negative	209 (60.8)	114 (59.4)	95 (62.5)	0.556
Positive	135 (39.2)	78 (40.6)	57 (37.5)	

G stage, *N* (%)				
0-1	32 (9.3)	20 (10.4)	12 (7.9)	0.057
2	176 (51.2)	105 (54.7)	71 (46.7)	
3-4	136 (39.5)	67 (34.9)	69 (45.4)	

F stage, *N* (%)				
0	26 (7.6)	17 (8.9)	9 (5.9)	0.000
1	84 (24.4)	55 (26.8)	29 (19.1)	
2	133 (38.7)	80 (41.7)	53 (34.9)	
3	68 (19.8)	35 (18.2)	33 (21.7)	
4	33 (9.6)	5 (2.6)	28 (18.4)	

Pathology staining, (%)				
Masson's trichrome staining (*N* = 157)	5.0 (2.2, 9.0)	3.1 (1.2, 7.0)	6.3 (3.1, 11.4)	0.001
Reticulin staining (*N* = 224)	2.3 (0.9, 4.5)	1.8 (0.7, 4.3)	2.9 (1.5, 5.0)	0.023

The data are expressed as median (25–75%). *P* for trends determined through the linear-by-linear association test. Liver histopathology of patients with G0, G1, G2, G3, and G4 according to the Scheuer scoring system. G1, portal inflammation; G2, mild piecemeal necrosis; G3, moderate piecemeal necrosis; G4, severe piecemeal necrosis and bridging necrosis. METAVIR scoring system. F0, no fibrosis; F1, portal fibrosis without septa; F2, septal fibrosis (portal-portal); F3, septal fibrosis (portal-central); F4, cirrhosis.

**Table 4:** Univariate and multivariate analyses producing odds ratio for significant F4 stage in chronic HBV patients.

Variables	Univariate analysis RR (95% C.I.)	*p* value	Multivariate analysis RR (95% C.I)	*p* value
AFP	1.02 (1.01–1.04)	0.001		
Alb	0.84 (0.77–0.92)	0.000	0.85 (0.74–0.97)	0.019
ALP	1.01 (1.00–1.01)	0.034		
ALT	1.00 (1.00–1.00)	0.016		
AST	1.00 (1.00–1.00)	0.010		
CHE	1.00 (1.00–1.00)	0.001		
CIV	1.01 (1.00–1.01)	0.001		
G	14.83 (4.51–48.76)	0.000	5.55 (1.56–19.67)	0.008
Hb	0.96 (0.93–0.99)	0.016		
Neutrophil	0.60 (0.40–0.88)	0.010		
PLT	0.98 (0.98–0.99)	0.001		
PTA	0.98 (0.96–0.99)	0.009		
RBC	0.16 (0.06–0.41)	0.000		
sGDNF	13.33 (4.76–37.34)	0.000	6.98 (1.10–17.94)	0.036
TBA	1.01 (1.00–1.02)	0.009		
TBil	1.04 (1.02–1.07)	0.001		
WBC	0.60 (0.43–0.86)	0.005		

All baseline covariates were included in the univariable analysis (two-sided *p* value < 0.05). Only covariates significantly associated with F4 in the univariable analysis (two-sided *p* value < 0.05) are shown and included in the multivariable model. Alb, albumin, AFP, alpha fetoprotein; ALP, alkaline phosphatase; ALT, alanine aminotransferase; AST, aspartate aminotransferase; CHE, cholinesterase; CIV, type IV collagen; Hb, hemoglobin; PLT, platelet count; PTA, prothrombin activity; RBC, red blood cell count; TBA, total bile acid; TBil, total bilirubin; WBC, white blood cell count.

**Table 5:** Areas under the receiver operating characteristic curve for the diagnostic accuracy of sGDNF, APRI, FIB-4, fibrotest, forn index, hepascore, and fibrometer for the diagnosis of histologic fibrosis stage F4.

	AUROC (95% CI)	Cutoff	Sensitivity (%)	Specificity (%)	PPV (%)	NPV (%)	Youden	vs. sGDNF *p* value
*Primary analysis F0–3 vs. F4*								
sGDNF	0.78 (0.73–0.82)	28.74	84.85	60.65	18.5	97.4	0.4550	
APRI	0.68 (0.63–0.73)	0.41	93.94	38.89	14.2	98.3	0.3283	0.1417
FIB-4	0.71 (0.66–0.76)	1.12	90.91	44.05	14.7	97.9	0.3496	0.3216
Fibrotest	0.70 (0.65–0.75)	0.89	63.64	70.10	18.4	94.8	0.3373	0.1998
Forn index	0.73 (0.68–0.78)	3.94	93.94	40.19	14.3	98.4	0.3413	0.4837
Hepascore	0.67 (0.62–0.72)	0.80	75.76	59.16	16.4	95.8	0.3492	0.1234
Fibrometer	0.69 (0.64–0.74)	2.54	75.76	56.91	15.7	95.7	0.3267	0.1856

*Second analysis F3 vs. F4*								
sGDNF	0.74 (0.65–0.83)	33.43	63.64	83.58	65.6	82.4	0.4722	
APRI	0.55 (0.44–0.65)	0.41	93.94	25.76	38.7	89.5	0.1970	0.0124
FIB-4	0.55 (0.44–0.65)	2.20	57.57	61.19	42.2	74.5	0.1877	0.0181
Fibrotest	0.61 (0.50–0.70)	0.89	63.64	59.70	43.8	76.9	0.2334	0.0802
Forn index	0.57 (0.47–0.67)	5.45	63.64	50.75	38.9	73.9	0.1438	0.0368
Hepascore	0.55 (0.45–0.65)	0.80	75.76	40.30	38.5	77.1	0.1606	0.0211
Fibrometer	0.53 (0.43–0.64)	1.69	87.88	25.37	36.7	81.0	0.1325	0.0133

AUROC of sGDNF vs. APRI, FIB-4, fibrotest, forn index, hepascore, fibrometer via DeLong test. NPV, negative predictive value; PPV, positive predictive value; Se, sensitivity; Sp, specificity. APRI, aspartate transaminase-platelet ratio index; FIB-4, fibrosis-4 index; APRI: (AST (U/L)/upper normal limit) × 100/platelets (10^9^/L). FIB-4 index: age (years) × AST (U/L)/(PLT (10^9^/L)) × (ALT (U/L)^1/2^). Fibrometer: −0.007 PLT (G/L) − 0.049 PI (%) + 0.012AST (U/L) + 0.005 *α*2M (mg/dL) + 0.021HA (*μ*g/L) − 0.270 urea (mmol/L) + 0.027 age (yr) + 3.718. Forn index: 7.811 − 3.131 × ln (PLT (10^9^/L)) + 0.781 × ln (GGT (U/L)) + 3.467 × ln (age) − 0.014 × (cholesterol (mg/dl). Hepascore: *y*/(*y*+1)*y* = exp (4.185818 − (0.0249 × age) + (0.7464 × sex) + (1.0039 × *α*2M g/L) + (0.0302 × HA *μ*g/L) + (0.0691 × bilirubin *μ*mol/L) − (0.0012 × GGT U/L)), male = 1, female = 0. Fibrotest: 4.467 × log (*α*2M (mg/dL)) − 1.357 × log Hap (g/L) + 1.017 log GGT (U/L) + 0.0281 × age + 1.737 × log TBil (*μ*mol/L) − 1.184 × apoA-I (g/L) + 0.301 × (sex) − 5.540, male = 1, female = 0.

## Data Availability

The datasets used and analyzed during the current study are available from the corresponding author on reasonable request.

## Abbreviations

GDNF: Glial cell line-derived neurotrophic factor
CHB: Chronic hepatitis B
ELISA: Enzyme-linked immunosorbent assay
AUROC: Area under the receiver operating characteristics curve
CI: Confidence interval
AST: Aspartate aminotransferase
FIB-4: Fibrosis index based on four factors
AFP: Alpha fetoprotein
Alb: Albumin
ALP: Alkaline phosphatase;
ALT: Alanine aminotransferase
ANOVA: Analysis of variance
APRI: Aspartate aminotransferase-to-platelet ratio index;
OR: Odds ratio
*γ*-GT: Gamma-glutamyl transferase
HBV: Hepatitis B virus
INR: International normalized ratio
IQR: Interquartile range
PCR: Polymerase chain reaction.

## Supplementary Materials

Supplementary Materials Supplemental methods: ELISA analysis and qPCR analysis. Supplemental Table 1: characteristics of patients related to sGDNF in chronic HBV patients. Supplemental Figure 1: study flow diagram and patient disposition. Supplemental Figure 2: mGDNF according to the histological grade and fibrosis stage.
