# Supplementary material for: Serum Glial Cell Line-Derived Neurotrophic Factor (sGDNF) Is a Novel Biomarker in Predicting Cirrhosis in Patients with Chronic Hepatitis B
Source: Can J Gastroenterol Hepatol. 2022 Jul 9;2022:1048104. doi: 10.1155/2022/1048104 (PMC9288342; doi:10.1155/2022/1048104)
Supplement: Supplementary Materials — Supplemental methods: ELISA analysis and qPCR analysis. Supplemental Table 1: characteristics of patients related to sGDNF in chronic HBV patients. Supplemental Figure 1: study flow diagram and patient disposition. Supplemental Figure 2: mGDNF according to the histological grade and fibrosis stage. [file 1048104.f1.docx]

**Supplemental materials**

**Serum** **glial cell line-derived neurotrophic factor (sGDNF) is a novel biomarker in predicting cirrhosis in patients with chronic hepatitis B**

**Supplemental Methods**

**ELISA analysis**

The human GDNF ELISA kit (cat no. DY212) from R&D Systems (MN, USA) was used. A 96-well microplate (R&D, cat no. DY990) was coated with the capture antibody. After washing with washing buffer and blocking for 1 h with reagent diluent, the samples were added and incubated for 2 h at 37 °C. The detection antibody was added and incubated for an additional 1 h at 37 °C. Streptavidin-horseradish peroxidase (HRP) was added and incubated for 20 min at 37 °C. Finally, the substrate solution was added and incubated for 20 min at 37 °C, followed by adding stop solution (2N H_2_SO_4_). Optical density was read at 450 nm using the Varioskan Lux (Thermo, MN, USA).

**qPCR analysis**

Total RNA was extracted using Trizol (Takara, Japan), followed by reverse transcription of total RNA to cDNA with a PrimeScript RT reagents kit (perfect real time) from Takara (Shanghai, China). Subsequently, cDNA was subjected to quantitative real-time polymerase chain reaction (PCR) with the ABI ViiA™ 7Dx real-time PCR system (Life Technologies, NY, USA). The levels of the target mRNAs were normalized to that of 18S rRNA (F-5’-GTAACCCGTTGAACCCCATT-3’; F-5’-CCATCCAATCGGTAGTAGCG-3’). The gene expression values are expressed as 2-ΔCt. Human GDNF primer sequences were provided by Sangon (Shanghai, China) and are as F-5’-GCAGTTAAGACACAACCCCG-3’; R-5’- AGTTATGGGATGTCGTGGCT-3’.

**Supplemental Table 1.** Characteristics of patients related to sGDNF in chronic HBV patients

|  | Total (N=344) | sGDNF-low (N=192) | sGDNF-high (N=152) | *p* value |
| --- | --- | --- | --- | --- |
| General information | |  |  |  |
| Age, yr | 40 $\pm$10 | 39 $\pm$10 | 40 $\pm10$ | 0.345 |
| Male, N (%) | 218 (63.4) | 122 (63.5) | 96 (63.2) | 0.942 |
| Liver-renal function | |  |  |  |
| ALT, U/L | 54 (29, 165) | 52.5 (29, 148) | 55 (30, 174) | 0.268 |
| AST, U/L | 41 (29, 83) | 39 (29, 79) | 47 (28, 90) | 0.215 |
| ALP, U/L | 108 (89, 136) | 106 (87, 132) | 113 (91, 139) | 0.220 |
| γ-GT, U/L | 50 (30, 110) | 48 (29, 107) | 54 (30, 125) | 0.350 |
| CHE, U/L | 7597$\pm2430$ | 7833$\pm2388$ | 7302±2464 | 0.127 |
| TBil, μmol/L | 17 (12, 23) | 16 (12, 21) | 17 (12, 26) | 0.244 |
| TBA, μmol/L | 11 (6, 23) | 11 (6, 19) | 11 (6, 34) | 0.160 |
| TP, g/L | 73$\pm6$ | 72$\pm6$ | 73$\pm7$ | 0.713 |
| Alb, g/L | 42 (39, 45) | 43 (40, 46) | 42 (38, 45) | 0.008 |
| BUN, mmol/L | 4.8$\pm1.3$ | 4.8$\pm1.4$ | 4.6$\pm1.1$ | 0.240 |
| Cr, μmol/L | 77 (70, 85） | 79 (71, 88） | 76 (69, 81） | 0.031 |
| Glucose-lipid |  |  |  |  |
| TC, mmol/L | 4.5 $\pm1.1$ | 4.6 ± 1.1 | 4.3 $\pm1.0$ | 0.062 |
| TG, mmol/L | 1.3 (0.9, 1.8） | 1.3 (1.0, 1.9） | 1.2 (0.9, 1.6） | 0.198 |
| HDL, mmol/L | 1.1 (0.9, 1.3） | 1.1 (0.9, 1.3) | 1.1 (0.9, 1.3) | 0.514 |
| LDL, mmol/L | 2.9$\pm$0.8 | $3.0\pm$0.9 | 2.8 $\pm$0.8 | 0.277 |
| Glucose, mmol/L | 4.8 (4.4, 5.4） | 4.9 (4.4, 5.3) | 4.8 (4.3, 5.5) | 0.639 |
| Blood cells |  |  |  |  |
| RBC, 10^12^/L | 4.9 $\pm0.48$ | 5.0 $\pm0.47$ | 4.8 $\pm0.48$ | 0.018 |
| Hb, g/L | 152 $\pm18$ | 153 $\pm12$ | 150 $\pm24$ | 0.353 |
| WBC, 10^9^/L | 5.7 $\pm$1.5 | 5.7 $\pm$1.5 | 5.6 $\pm$1.6 | 0.541 |
| Neutrophil, 10^9^/L | 3.0 $\pm1.2$ | 3.0 $\pm1.2$ | 3.0 $\pm1.1$ | 0.838 |
| PLT, 10^9^/L | 178 $\pm$56 | 184 $\pm$47 | 171 $\pm$64 | 0.090 |
| Virus |  |  |  |  |
| HBV DNA, (log10, IU/ml) | 4.7 (2.7, 7.2） | 4.7 (2.7, 7.1) | 4.9 (2.8, 7.5) | 0.652 |
| HBsAg, (log10, ng/ml) | 2.6 (2.2, 2.7) | 2.6 (2.1 2.7) | 2.5 (2.3, 2.7) | 0.912 |
| Fibrotic factor |  |  |  |  |
| HA, ng/ml | 65 (34, 108) | 67 (35, 102） | 57 (32, 123） | 0.894 |
| PCⅢ, ng/ml | 7.5 (5.5, 11.4) | 6.7 (5.1, 10.0） | 8.5 (5.7, 14.3) | 0.012 |
| CⅣ, ng/ml | 47 (30, 80) | 43 (31, 74) | 47 (29, 99) | 0.315 |
| LN, ng/ml | 48 (25, 71) | 44 (22, 62) | 52 (29, 100) | 0.047 |
| sGDNF, pg/ml | 28 (26, 32) | 27 (25, 27) | 32 (29, 44) | 0.000 |
| Coagulation-related parameters and other | |  |  |  |
| PT, s | 13 $\pm1.3$ | 13$\pm1.2$ | 13 $\pm1.3$ | 0.491 |
| INR | 1.04 (0.98,1.09) | 1.02 (0.98, 1.09) | 1.05 (0.98, 1.12) | 0.179 |
| PTA, % | 90 $\pm$23 | 91 $\pm$24 | 90 $\pm$ 22 | 0.770 |
| AFP, ng/ml | 3.6 (2.3, 8.1) | 3.4 (2.5, 6.4) | 4.8 (2.3, 12.2) | 0.125 |

The data are expressed as the mean ± standard deviation or median (25-75%)

AFP, alpha fetoprotein; Alb, albumin; ALP, alkaline phosphatase; ALT, alanine aminotransferase; AST, aspartate aminotransferase; BUN, blood urea nitrogen; CHE, cholinesterase; Cr, creatinine; γ-GT, γ-glutamyl transpeptidase; HA, hyaluronic acid; Hb, hemoglobin; HBsAg, hepatitis B surface antigen; HBV-DNA, hepatitis B virus-DNA; HDL, high-density lipoprotein; TC, Total cholesterol; INR, international normalized ratio; CIV, type IV collagen; LN, Laminin; PCIII, type III procollagen; PLT, platelet count; RBC, red blood cell; TBA, total biliary acid; TBil, total bilirubin; TC, total cholesterol; TG, triglyceride; TP, total protein; PTA, prothrombin activity; WBC, white blood cell count.

**Supplemental Figures and Figure Legends**

**
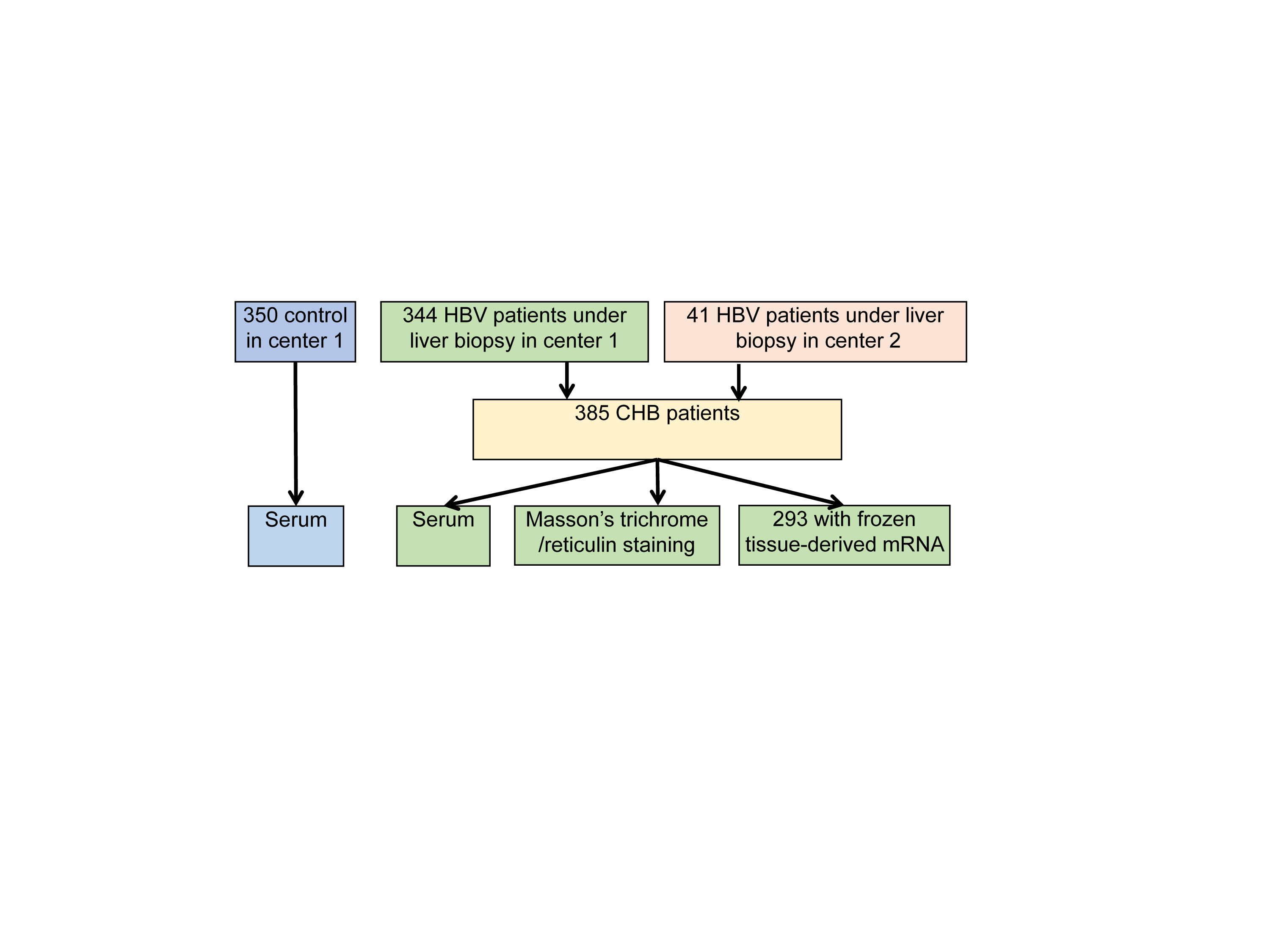
**

**Supplemental Figure 1. Study flow diagram and patient disposition.**

**
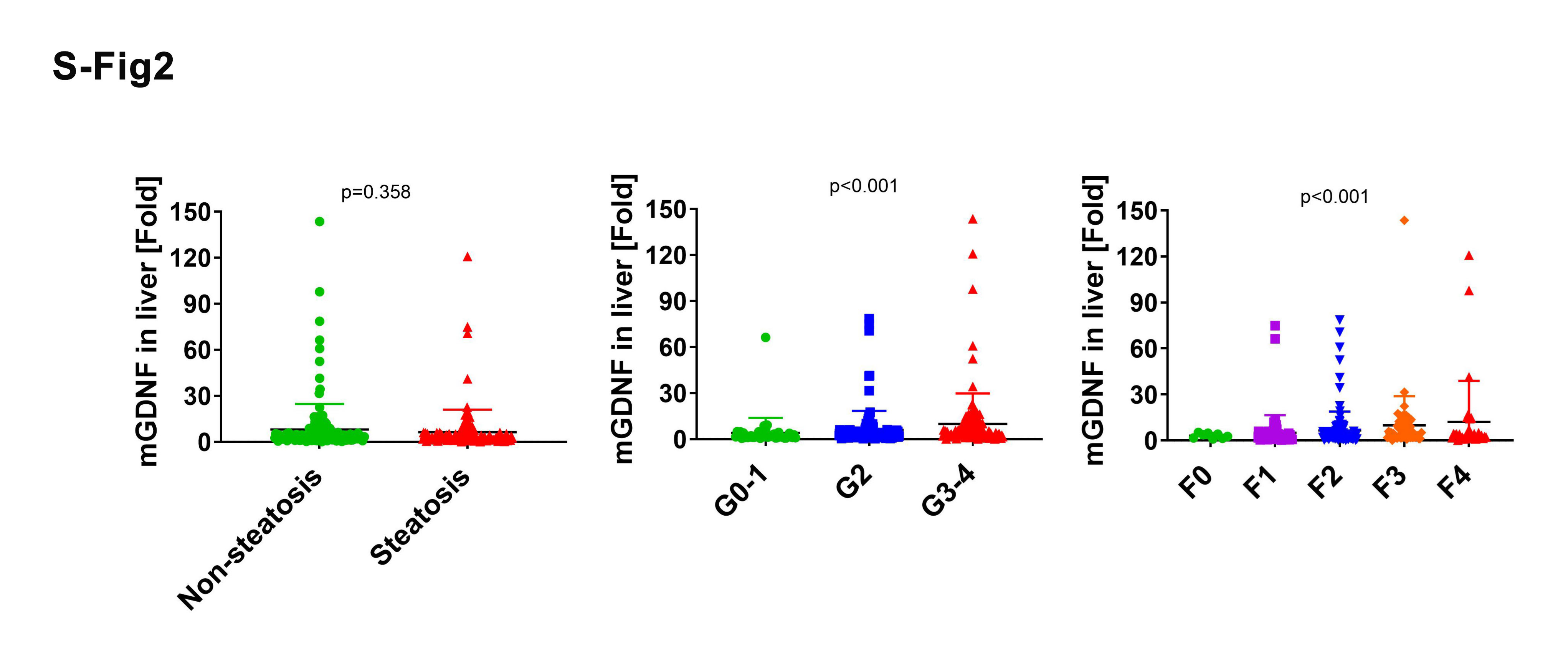
**

**Supplemental Figure 2 mGDNF according to the histological grade and fibrosis stage**

mGDNF expression according to the histological grade and fibrosis stage in 293 patients. Liver histopathology of patients with G0, G1, G2, G3, and G4 according to the Scheuer scoring system. G1, portal inflammation; G2, mild piecemeal necrosis; G3, moderate piecemeal necrosis; G4, severe piecemeal necrosis and bridging necrosis. METAVIR scoring system. F0, no fibrosis; F1, Portal fibrosis without septa; F2, Septal fibrosis (portal-portal); F3, Septal fibrosis (portal-central); F4, Cirrhosis.
